# Surgical treatment of refractory epilepsy after chemotherapy in two children with leukemia^☆^

**DOI:** 10.1016/j.ebcr.2012.12.001

**Published:** 2013-02-04

**Authors:** Yun Leng, Tao Yu, Yongjie Li, Wenming Chen

**Affiliations:** a Department of Hematology, Chaoyang Hospital of Capital Medical University, Beijing, China; b Beijing Institute of Functional Neurosurgery, Xuanwu Hospital, Capital Medical University, Beijing, China

**Keywords:** Acute leukemia, Chemotherapy, Refractory epilepsy, Surgery

## Abstract

Refractory epilepsy is a rare, long-term complication in children with acute leukemia who are receiving chemotherapy. A few studies have reported cases of several patients who developed recurrent complex partial seizures after the initiation of chemotherapy. In these cases, the cause of the refractory seizures was identified as mesial temporal lobe sclerosis. Here, we report on two patients with extratemporal lesions accompanied by refractory seizures, a long-term complication of acute lymphocytic leukemia. Using presurgical evaluations and measures of the surgical outcomes, the lesions were identified as epileptogenic and were located in the mesial temporal lobe. The underlying pathophysiological background is discussed to aid in understanding this uncommon long-term complication.

## 1 Introduction

Epileptic seizures are a complication of chemotherapy in children diagnosed with acute leukemia. Epilepsy develops in 10% to 13% of children with acute lymphocytic leukemia (ALL) [1], [2]. However, medically refractory epilepsy as a late complication has rarely been reported, and the mechanism of intractable seizures after chemotherapy in children with leukemia has not been clearly explained. We present two pediatric patients with ALL who developed medically refractory epilepsy following chemotherapy. These two patients were diagnosed with temporal ‘plus’ epilepsies and were successfully treated by surgical resection of the epileptogenic zone. The underlying pathophysiological background and the treatment for this complication are discussed.

## 2 Case 1

A 10-year-old boy was admitted to the hospital with pre-B acute lymphocytic leukemia. He received CVDLP (cyclophosphamide, vincristine, daunomycine, l-asparaginase, prednisone) and achieved complete remission after one cycle of chemotherapy. However, coagulation abnormalities were detected with the third dose of l-asparaginase. He developed cerebral dysfunction with confusion and convulsions. He was diagnosed with a cerebral parenchymal hemorrhage of the left parietal–occipital lobe and an epidural hemorrhage of the left occipital region using a cranial computed tomography (CT) scan. After a plasma transfusion, the hemorrhage was gradually absorbed. He then received an intrathecal injection of MTX (methotrexate) and Ara-c (cytarabine). Next, the patient was alternately treated with VM26 (teniposide), Ara-c, HD-MTX (high dose methotrexate), and VDLP (vincristine, daunorubicin, l-asparaginase, prednisone).

**Fig. 1:**
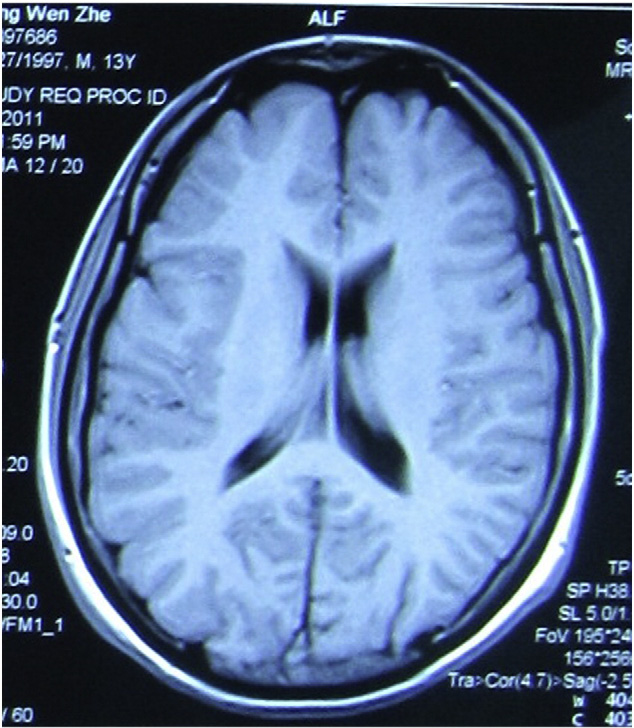
Slight atrophy of the cortex in the left temporal–parietal region.

**Fig. 2:**
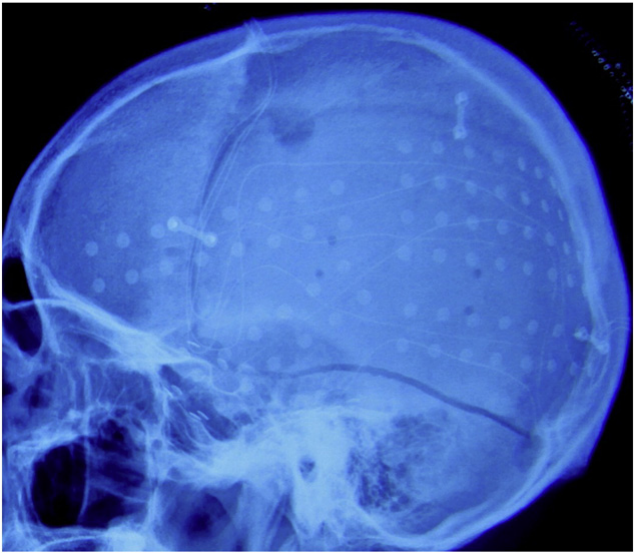
Cortical electrodes were implanted.

Four years after the discontinuation of the antileukemic treatment, the patient had his first epileptic seizure. From then on, he experienced stereotyped seizures that lasted 2 to 3 min. The seizures were preceded by an aura that the patient described as a feeling of needing to urinate. During the seizures, the patient sat up and purposelessly moved his body, including movements of oral automatism, eye blinks, and tonic and clonic movements of his right face and right limbs. The seizures mostly occurred during sleep but occasionally occurred during the daytime. Several antiepileptic drugs, including topiramate, clonazepam, phenytoin sodium, carbamazepine, lamotrigine, and sodium valproate, were administered in the following years, but the outcome was always disappointing. When he was admitted to the hospital, his seizures were occurring several times per week. Interictal scalp electroencephalography (EEG) recordings revealed left temporal–occipital discharges, and long-term video-EEG monitoring revealed that the seizures originated from the same region. Magnetic resonance imaging (MRI) scans showed slight atrophy of the cortex in this region (Fig. 1). Long-term intracranial recording was performed to localize the epileptogenic focus and to more accurately map the functional cortex (Fig. 2). The interictal cortical EEG showed repeated spikes in the left temporal lobe, especially in the posterior temporal region. The ictal EEG showed that the seizures predominantly originated from the posterior part of the temporal lobe. Surgical excision of the epileptogenic tissue in the left temporal–parietal region was performed. The patient was seizure-free for the following two months. However, the seizures gradually reappeared two months after the operation. Six months later, after further preoperative evaluation, an additional resection was performed to remove the left anterior temporal lobe (4 cm in length), including 2 cm of the hippocampus. Since the operation, the patient has been seizure-free for over one year and is medicated with carbamazepine and lamotrigine. Postoperative histopathological examination revealed cicatrix and gliosis in the resected posterior temporal tissue (first resection) and neuronal loss and gliosis in the hippocampus (second resection).

## 3 Case 2

**Fig. 3:**
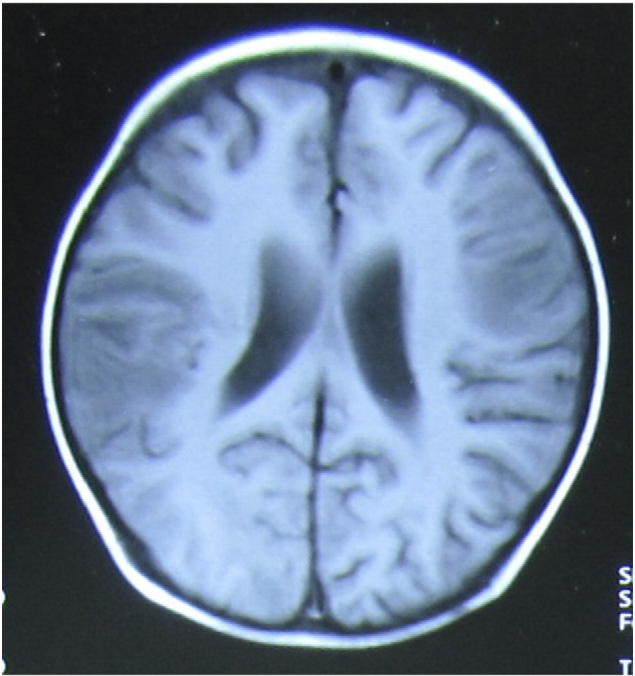
Infiltrated lesions in the right parietal and left frontal lobes.

A 3-year-old boy was admitted to the hospital with ALL (L1). He received DVLP (daunomycine, vincristine, l-asparaginase, prednisone) and achieved complete remission after one cycle of treatment. However, he developed recurrent seizures approximately one month after his final chemotherapy cycle. An MRI scan showed infiltrated lesions in his brain (Fig. 3). At that time, he received a chemotherapy protocol of VM26, Ara-c, HD-MTX, and VDLP. His seizures gradually decreased and finally disappeared with elimination of the lesions and effective chemotherapy without antiepileptic drugs. However, his seizures reoccurred and increased in frequency two years later. Sometimes the seizures occurred after an aura of abdominal discomfort. During the seizures, the patient had automatisms of his right hand and arm and then displayed tonic and clonic movements in his left face and left limbs. The seizures gradually failed to respond to antiepileptic drugs, including topiramate, carbamazepine, sodium valproate, and oxcarbazepine, over the following years. The frequency of his seizures ranged from 20 times/day to once a week.

**Fig. 4:**
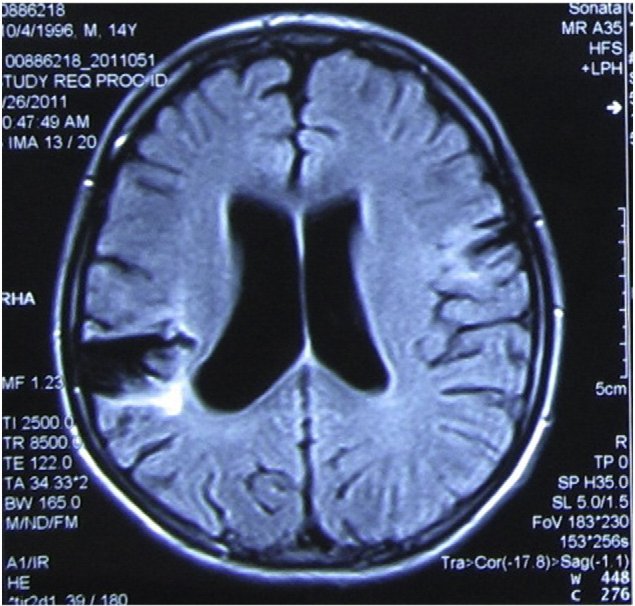
Lesions in the right posterior temporal–parietal region.

**Fig. 5:**
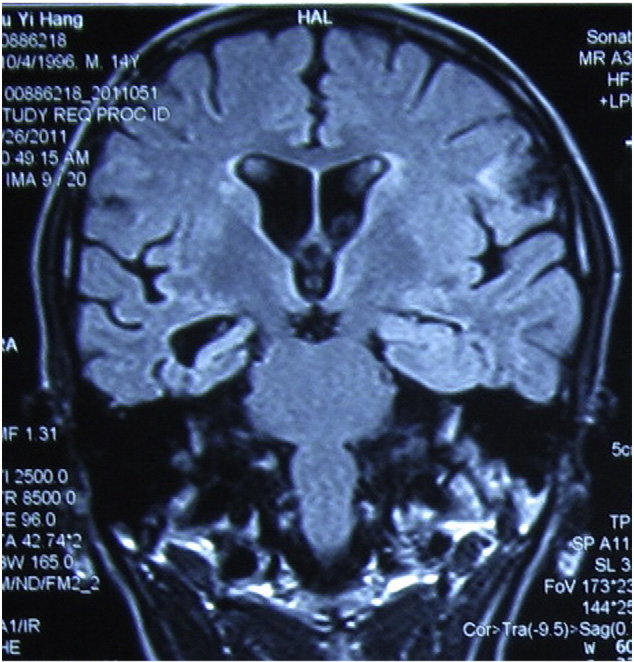
Mesial temporal lobe sclerosis in the right temporal lobe.

His medically refractory epilepsy was surgically evaluated when he was fifteen years old. Interictal scalp EEG recordings revealed right temporal–parietal discharges, and long-term video-EEG monitoring revealed that the seizures originated from the same region. Magnetic resonance imaging scans also showed hippocampal sclerosis on the right side, in addition to the lesion in the right temporal–parietal region (Figs. 4 and 5). Furthermore, the spike dipoles of the magnetoencephalogram were concentrated around the lesion. Therefore, the epileptogenic zone (EZ) was localized in the region of the posterior temporal-parietal lobe. Surgical resection was performed. The resection included both the hippocampus and the posterior temporal lesion. A postoperative histopathological examination revealed severe hippocampal sclerosis and cicatrix gyrus in the resected temporal–parietal tissue. The patient has been seizure-free in the year following the operation and uses the medication oxcarbazepine.

## 4 Discussion

Although epileptic seizures are relatively common in children with acute leukemia, refractory seizures are rare. A few studies have reported several cases of recurrent complex partial seizures (CPSs) that developed after the initiation of chemotherapy. Goyal described three children with acute leukemia who developed refractory CPSs after chemotherapy and cranial irradiation [3]. Additionally, Faraci reported on the cases of three children who underwent allogenic hematopoietic stem cell transplantation with cyclosporin-A and developed repeated seizures [4]. Other cases have been described in recent years. The reason for the repeated seizures in all of these patients was identified as mesial temporal lobe sclerosis (MTS). Various factors play a role in the development of MTS. Diffuse cerebral atrophy, leukoencephalopathy, central pontine myelinolysis, leukemic infiltration via the arachnoids, and microangiopathy are related to the primary effects of the disease and/or the treatment [5], [6], [7], [8], [9]. The use of chemotherapeutic agents, such as systemic vincristine, l-asparaginase, systemic or intrathecal methotrexate, and cytosine arabinoside might result in disturbances of brain metabolism and the vascular system. Furthermore, the use of cranial radiotherapy could cause white matter destruction, vascular damage leading to hemorrhage and calcification, and enlargement of the ventricles and/or sulci [10], [11], [12].

However, the two patients described in this report were diagnosed with temporal plus epilepsy rather than with classical temporal lobe epilepsy [13]. The focal extratemporal cortex was involved in the EZs located next to the sclerosis in the mesial temporal lobe. The underlying pathophysiological background of this complication was worthy of discussion to explore the mechanisms of the development of refractory epilepsy.

In the first patient, the hematoma that presented after chemotherapy damaged some areas of the focal cortex, which was verified by the neuronal loss and fibrous gliosis observed in the histopathological exam. The scar lesion may have altered the balance of excitation and inhibition in the cortex in the years following chemotherapy and may be the origination point of the seizures. However, the predominate reason for medically resistant seizures may be secondary MTS because only when the mesial temporal lobe was removed by the second operation was the patient continuously seizure-free. In the second patient, all the evaluations identified the posterior temporal–parietal lesion as epileptogenic, in addition to the patient having MTS.

Medically refractory epilepsy should be considered as a potential late complication in children with acute leukemia who are receiving chemotherapy. Any focal damage caused by the leukemic infusion, chemotherapy, radiotherapy, or viral infection might contribute to the development of long-term seizure events. The cortical lesions and the mesial temporal lobe should be examined in follow-up visits. Repeated EEG monitoring might be helpful for some of these patients in predicting the possibility of recurrent seizures. Furthermore, to our limited knowledge, surgery is a potential treatment for some patients with medically refractory epilepsy. Although it is more difficult to localize the epileptogenic zone in patients with temporal ‘plus’ epilepsies, perfect outcomes could be expected after carefully planned operations [14].
